# SPECT Imaging of Epilepsy: An Overview and Comparison with F-18 FDG PET

**DOI:** 10.1155/2011/813028

**Published:** 2011-07-14

**Authors:** Sunhee Kim, James M. Mountz

**Affiliations:** ^1^Division of Nuclear Medicine, Department of Radiology, Children's Hospital of Pittsburgh and The University of Pittsburgh Medical Center, Pittsburgh, PA 15213, USA; ^2^Division of Nuclear Medicine, Department of Radiology, University of Pittsburgh Medical Center, PET Facility, B-932, 200 Lothrop Street, Pittsburgh, PA 15213, USA

## Abstract

Epilepsy surgery is highly effective in treating refractory epilepsy, but requires accurate presurgical localization of the epileptogenic focus. Briefly, localization of the region of seizure onset traditionally dependents on seizure semiology, scalp EEG recordings and correlation with anatomical imaging modalities such as MRI. The introduction of noninvasive functional neuroimaging methods, including single-photon emission computed tomography (SPECT) and positron emission tomography (PET) has dramatically changed the method for presurgical epilepsy evaluation. These imaging modalities have become powerful tools for the investigation of brain function and are an essential part of the evaluation of epileptic patients. Of these methods, SPECT has the practical capacity to image blood flow functional changes that occur during seizures in the routine clinical setting. In this review we present the basic principles of epilepsy SPECT and PET imaging. We discuss the properties of the SPECT tracers to be used for this purpose and imaging acquisition protocols as well as the diagnostic performance of SPECT in addition to SPECT image analysis methods. This is followed by a discussion and comparison to F-18 FDG PET acquisition and imaging analysis methods.

## 1. Introduction

Epilepsy surgery can be highly effective in treating refractory epilepsy if performed in properly selected patients with well-delineated ictal foci [1]. The greatest challenge is accurate localization, but only a small fraction of the patients whose epilepsy becomes refractory ultimately receive surgery. In the past, localization of the region of seizure onset was dependent upon scalp, cortical, and depth electroencephalography (EEG). However, scalp EEG has disadvantages such as dependency on cortical surface effects and low spatial resolution that can lead to mislocalization of epileptogenic foci. Both cortical and depth EEG have a limited spatial sampling area that is confined to regions accessible by electrode placement. Depth EEG can detect signals from deeper structures, but it is more invasive, which can lead to surgical complications [2]. 

The introduction of noninvasive neuroimaging methods, such as single-photon emission computed tomography (SPECT), positron emission tomography (PET), and magnetic resonance imaging (MRI), has dramatically changed presurgical epilepsy evaluation. These imaging methods have become powerful tools for the investigation of brain function and an essential part of the evaluation of epileptic patients. Of these methods, only SPECT has the practical capacity to image blood flow functional changes that occur during seizures in the routine clinical setting. Although functional MRI (fMRI) could, in theory, be used for this purpose, it is impractical due to patient movement during most types of seizures, a problem that is overcome by the timing and technique of SPECT imaging. 

In this paper, we review basic principles of epilepsy SPECT, SPECT tracers, imaging acquisition, the diagnostic performance of SPECT, and imaging analysis methods. This is followed by a discussion and comparison to PET tracer acquisition methods and imaging analysis methods.

## 2. Central Nervous System Radiopharmaceuticals for SPECT

SPECT radiopharmaceuticals used for measuring regional cerebral blood flow (rCBF) are lipophilic agents which are transported from the vascular compartment to the normal brain tissue compartment by diffusion and are distributed proportionally to regional tissue blood flow. After this first phase of transport (during the first pass through the brain), the tracer is essentially irreversibly trapped in the tissue compartment and does not change its relative distribution over time. These properties are essential in ictal SPECT, since the tracer is essentially trapped during the first few seconds after injection and maintains that distribution for hours, allowing the patient to be stabilized and imaged at rest, but the emission of photons still reflects the tracer distribution pattern seconds after injection. The two major blood flow agents used in brain SPECT imaging are technetium-99m hexamethyl-propylene amine oxime (Tc-99m HMPAO) and Tc-99m ethyl cysteinate dimer (Tc-99m ECD) [3, 4].

### 2.1. Tc-99m Hexamethyl-propylene Amine Oxime (HMPAO)

To understand the uptake mechanism of Tc-99m hexamethyl-propylene amine oxime (Tc-99m HMPAO), a three-compartment analysis model can be used for analysis [5]. In this model the first compartment is the lipophilic tracer in the blood pool of the brain, but outside of the blood brain-barrier. The second compartment is comprised of the lipophilic tracer inside of the blood brain-barrier. The third compartment is the hydrophilic form of the tracer that is retained in the brain. Transport from the first compartment to the second compartment represents efflux of lipophilic tracer from the blood compartment to the brain compartment. Back-exchange from the third compartment to the second compartment represents back-diffusion of the lipophilic form of the tracer and is essentially equal to zero since the tracer is irreversibly trapped (by intracellular reaction with glutathione) in the brain.

### 2.2. Tc-99m Ethyl Cysteinate Dimer (ECD)

**Figure 1:**
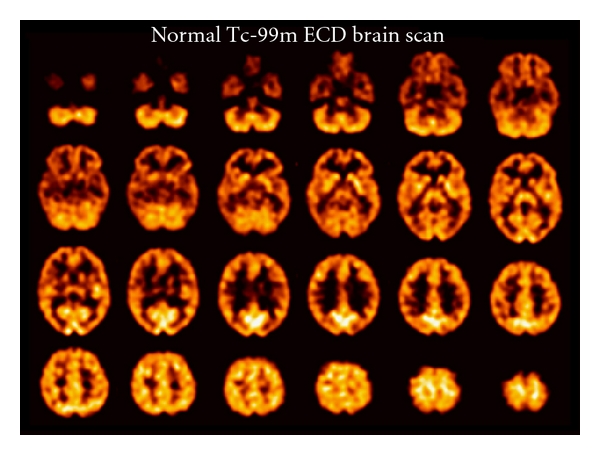
Transverse tomographic images from a normal 41-year-old female subject after injection of 20 mCi Tc-99m ECD. The transverse images are arranged parallel to and sequentially above the canthomeatal line, with the cerebellum at the top left and the vertex of the brain at the bottom right. The scan thickness is 4 mm. The scan resolution is approximately 7 mm full width at half maximum.

The second tracer commonly used in brain SPECT to measure regional cerebral perfusion is Tc-99m ECD [6]. This radiopharmaceutical is lipophilic like Tc-99m HMPAO and rapidly traverses the endothelium and capillary membranes into the brain cells. However, in the third compartment irreversible trapping mechanism of this tracer differs from Tc-99m HMPAO, since Tc-99m ECD is enzymatically metabolized to a polar complex, which is trapped in the brain. This tracer has been reported to demonstrate less nonspecific scalp and facial tissue background activity compared with Tc-99m HMPAO.  Figure 1 shows a normal Tc-99m ECD brain SPECT scan after injection of 20 mCi (740 MBq) I.V.

### 2.3. Differences in Cerebral Distribution of Tc-99m HMPAO and Tc-99m ECD

Several studies reported higher extracerebral background activity with Tc-99m HMPAO than with Tc-99m ECD due to the slower clearance rate of Tc-99m HMPAO from the blood [7–10]. This can contribute to a lowering of the cortical uptake to background ratio with subsequent decreased detectability of epileptogenic focus. Additionally, Tc-99m HMPAO tends to underestimate the high flow rate due to a nonlinear extraction pattern to the flow whereas Tc-99m ECD reflects rCBF more linearly than Tc-99m HMPAO at higher flow rates [11]. Therefore, Tc-99m ECD may be more sensitive in detecting a hyperperfused epileptogenic focus at a high flow rate.

### 2.4. Comparison of Radiopharmaceutical Diagnostic Performance of Tc-99m HMPAO versus Tc-99m ECD

Lee et al. demonstrated that Tc-99m HMPAO has a similar localization rate (82%) to that of Tc-99m ECD (71%) in patients with TLE (in this group a total of 17 patients had Tc-99m HMPAO SPECT and 7 patients had Tc-99m ECD SPECT), with a higher degree of hyperperfusion in the Tc-99m HMPAO group [12]. For patients with neocortical epilepsy (23 patients had Tc-99m HMPAO SPECT and 7 patients had Tc-99m ECD SPECT), there was a higher localization rate (70% versus 29%) and degree of hyperperfusion in the Tc-99m HMPAO group. The authors summarized that the sensitivity of Tc-99m ECD ictal SPECT is similar to that of Tc-99m HMPAO ictal SPECT in TLE. However, ictal hyperperfusion was higher with the Tc-99m HMPAO SPECT in patients with neocortical epilepsy. Tc-99m HMPAO ictal SPECT also was superior to Tc-99m ECD ictal SPECT in sensitivity and degree of hyperperfusion. They concluded that the diagnostic performance and contrast of hyperperfused areas at the epileptogenic zones of Tc-99m HMPAO ictal SPECT were better than those of the Tc-99m ECD ictal SPECT in their group of patients.

## 3. Image Acquisition

### 3.1. SPECT Image Acquisition

In-single photon emission computed tomography (SPECT) of the brain, dual and triple-head Anger gamma cameras are now in common use and can provide very high resolution images (approximately seven millimeters full width half maximum (FWHM) extrinsic resolution) [13]. The resolution has improved, primarily, due to the increased count detection capability of these cameras. In addition, these cameras allow faster throughput of patients since the scan time can be decreased. Scanning can be performed in temporal segments, with summation of the projection images at the end of acquisition. This enables salvaging of studies in which patient motion might occur. For example, a 30-minute scan can be divided into two 15-minute segments, each obtaining a 360° set of projection images. If the patient moves during the last 15-minute imaging segment, the first 15-minute imaging segment can be used for reconstruction of the complete set of tomographic images.

### 3.2. Ictal SPECT: Practical Issues

In order to perform these studies, Tc-99m HMPAO must be readily available at the patient's bedside allowing for rapid injection by a trained technologist or other personnel immediately available at the time of seizure onset. The ictal injection should be performed in a rapid bolus fashion such that the entire tracer is injected during the first onset of seizure (ictus). The patient is then sedated and transferred to the SPECT scanner within several hours to acquire the brain SPECT scan which will indicate the regional cerebral perfusion at the time of ictus. This method is feasible since Tc-99m HMPAO is irreversibly trapped in the epileptogenic hyperemic region at the time of seizure, and during the period between injection and scan, there is essentially no redistribution. The subsequent scan (albeit several hours after the injection) will still show hyperemia (increased tracer counts) in the region of the epileptogenic focus.

## 4. Diagnostic Performance of Ictal and Interictal SPECT

**Figure 2:**
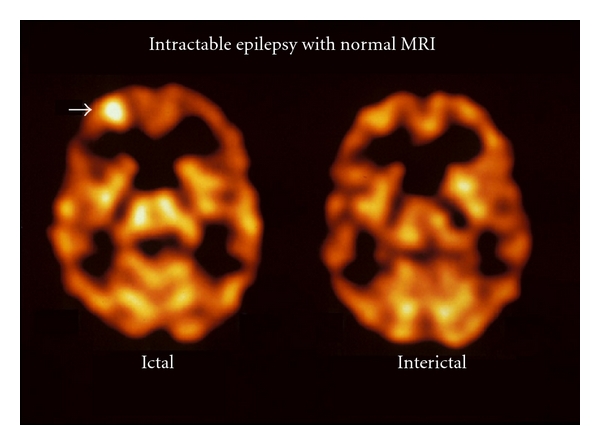
Illustration of the value of ictal SPECT in a 38-year-old male with intractable epilepsy. The MRI was normal. The Tc-99m HMPAO ictal brain SPECT scan showed a focal area of hyperperfusion in the right frontal area. In this case, the interictal scan shows normal perfusion to this area of the brain.

The sensitivity of ictal SPECT is theoretically higher than that of interictal SPECT because of the large CBF increase from the baseline that occurs during the ictal phase [14]. This is a substantially larger perfusion difference (approximately 50%) than from the 0–10% to at most 40% reduction of flow seen during the interictal phase [15, 16]. A comparison between ictal and interictal SPECT is shown in Figure 2. Several prior studies have shown a difference in sensitivity between ictal and interictal SPECT [17]. Overall, the highest sensitivity was seen in ictal SPECT (97% to 100%), followed by postictal SPECT (75% to 77%). Interictal SPECT was shown to have the lowest sensitivity (43% to 44%).

**Figure 3:**
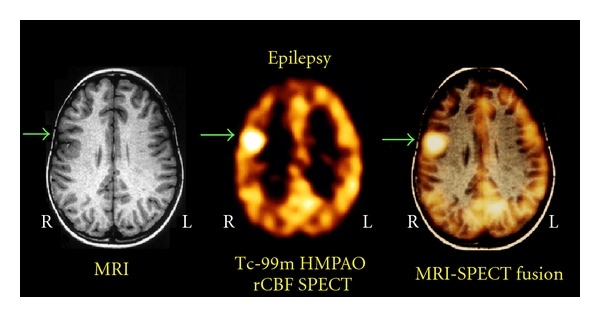
Tc-99m HMPAO ictal brain SPECT scan section (middle) showing a focal area of hyperperfusion in the right premotor area. The right to left asymmetry in blood flow for this region was 1.32, and the intensity of uptake in the right frontal lobe measured 1.13 (cortical to cerebellar ratio) with a range of normals = mean ± 1SD of 0.90 ± 0.07. The ictal brain SPECT scan was subsequently coregistered with the MRI scan (left). The resulting fusion image (right) shows the anatomic location of the epileptogenic focus, which was surgically excised, and the patient was rendered seizure-free.

Figure 3 illustrates the value of ictal SPECT in a nine-year-old right-handed boy who had a seven-year history of intractable seizures. The figure shows a Tc-99m HMPAO brain SPECT scan which was performed 2 hours after tracer injection. The tracer was injected at the bedside 3 seconds after seizure onset (the seizure lasted ~25 seconds). The ictal SPECT scan shows a focal area of intense uptake in the right frontal lobe. Computed tomography (CT) and magnetic resonance imaging (MRI) studies carried out at our and other institutions were normal. Multiple EEG investigations were inconclusive. Previous EEGs revealed infrequent slowing over the right hemisphere. Multiple video EEG monitoring studies performed at our and other institutions showed stereotypical seizures with no ictal scalp localization. Interictal activity revealed occasional sharp discharges involving the right frontal central parietal regions.

## 5. Imaging Analysis and Interpretation

### 5.1. Methods of Ictal-Interictal SPECT Subtraction

The traditional qualitative visual analysis of a SPECT perfusion study involves comparison of each cerebral region with the contralateral side. Limitations of interpretation of SPECT by conventional visual assessment include difficulty in the detection of subtle changes that can be associated with inhomogeneous baseline perfusion patterns, variation in the amount of injected radioisotope, time of injection, and patient positioning [18]. Also, the interpretation can be subjective, depending on the readers, which can create interobserver and even intraobserver variability. In addition, if the epileptogenic zone is hypoperfused at baseline (interictal), the ictal increase in tracer uptake may be obscured (i.e., appear normal) despite relative hyperperfusion [19]. 

**Figure 4:**
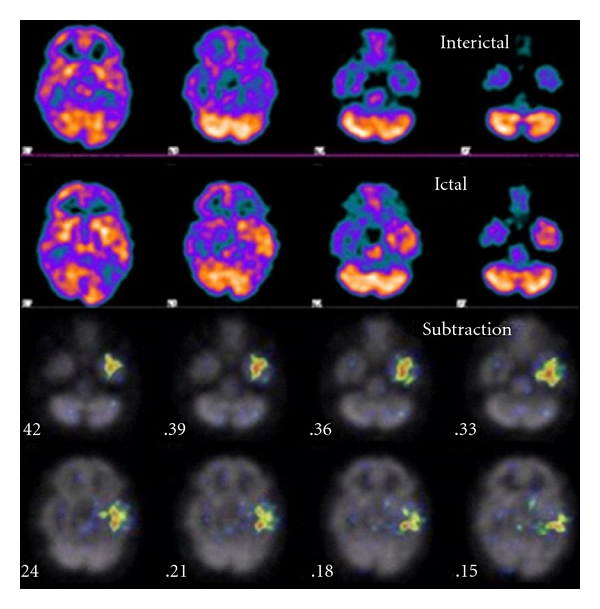
An interictal technetium-99m ECD brain SPECT scan (top) as compared with an ictal technetium-99m ECD brain SPECT scan (second row). On the ictal brain SPECT scan one can see increased hyperemia involving the left temporal lobe (third and fourth rows). Using subtraction comparison, one can see statistically significant differences between the ictal and interictal scan involving the left temporal lobe. The test for statistical significance has z values between three and four (red color = z value of 4).

For a more objective and quantitative analysis, Zubal et al. proposed a subtraction method in which coregistered ictal and interictal images were normalized based on total pixel counts in the brain and then subtracted from each other [20]. Therefore, each pixel represented the percent difference between two data sets (ictal and interictal). Overall, subtraction analysis had a higher concordance rate with the outcome “gold standard” (successful surgical outcome or intracranial EEG) compared with conventional side-by-side visual analysis of ictal (or peri-ictal) and interictal SPECT [21–24]. Another subtraction method proposed by O'Brien et al. is SISCOM (subtraction ictal SPECT coregistered to MRI) which also demonstrates similar results with a significantly higher concordance rate than visual analysis [19]. This includes the following steps: (1) SPECT to SPECT coregistration with distance-based surface matching technique, (2) SPECT normalization, (3) SPECT subtraction and thresholding, and (4) subtraction SPECT to MRI coregistration [19].  Figure 4 shows ictal-interictal subtraction images from a 39-year-old male with a history of refractory complex partial epilepsy for 25 years. His MRI scan has been consistently normal. Interictal SPECT showed hypometabolism of the left temporal region while ictal SPECT showed increased rCBF in both the lateral and mesial left temporal lobe. Subtraction analysis clearly showed that there was increased left temporal rCBF (*z* score >+3).

### 5.2. Statistical Parametric Mapping (SPM)

Statistical parametric mapping refers to the construction and assessment of spatially extended statistical processes used to test hypotheses about functional imaging data. In rCBF SPECT or F-18 FDG PET image data analysis, this translates to methods to test hypotheses about regionally specific effects (e.g., the probability of finding a region of increased regional cerebral perfusion or metabolism by chance). When two image data sets are evaluated by SPM, all voxels contained within the scans are compared in the same space on a voxel-by-voxel basis using linear constraints to test hypotheses for specific focal effects using a univariate statistical test. The resulting statistical parameters are then assembled onto an image (i.e., the statistical parametric map). Differences of one map compared with the map derived from the other scans are interpreted as regionally specific effects, attributable to some alteration in brain function from one scan to the other. The significance of these differences is assessed using statistical tests (usually the *t* or *F* statistic). Criteria for accepting voxels (those intended to represent true changes in regional cerebral perfusion) can be set for voxel height (*p*) and extent of contiguous cluster of voxels (*k*). For visualization of the results, a pseudocolor scale can be applied to accepted significant voxels, which are then overlaied in a semitransparent fashion onto the MRI of either the normative atlas or the patient's own MRI anatomy. The most recent version of SPM (SPM2) combines the general linear model to create the statistical map and the random field theory to make statistical inference about regional effects. Software for SPM analysis is available as Freeware from the Welcome Department of Imaging Neuroscience (University College London, London, UK). Although the SPM package includes most of the programs required for image processing and analysis, visualization of images and some processing or image editing and reformatting may require more dedicated biomedical image processing software. 

The use of SPM image analysis is now increasingly being applied in the clinical diagnosis of neuroimaging of numerous disorders including epilepsy. An ictal SPECT scan can be compared with the interictal SPECT scan and correlated with a normal brain SPECT atlas using SPM to identify regions of significant alterations in regional cerebral blood flow related to seizure activity and localize these regions in Montreal Neurological Atlas space. If regions of maximal ictal cerebral blood flow can be reliably identified by this method, fully objective ictal rCBF SPECT analysis can be made widely available for clinical use. Recent studies support SPM analysis of ictal SPECT scans [25]. 

## 6. The Role of F-18 FDG PET in the Evaluation of Epilepsy

### 6.1. A Second Major Class of Radiopharmaceuticals Are Those That Measure Brain Metabolism

**Figure 5:**
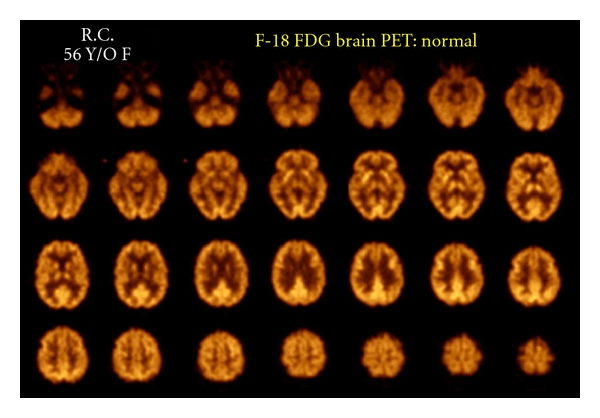
Normal F-18 FDG PET image from a 56-year-old female subject at rest. PET scan image slice thickness of ~4 mm reconstructed in plane image resolution = 5 mm FWHM.

These radiopharmaceuticals are transported to the brain tissues by regional cerebral blood flow, but subsequent regional cerebral distribution reflects the utilization rate of the tracer in a cerebral metabolic pathway. The PET radiopharmaceutical predominantly used is F-18 fluorodeoxyglucose (F-18 FDG) [26].  Figure 5 illustrates a normal F-18 FDG PET scan.

### 6.2. F-18 FDG PET Image Acquisition

Approximately 10 mCi F-18 FDG I.V is injected. The tomograph should be of the latest generation, multislice to cover the entire brain. The 3D acquisition mode should be used to accommodate lower dosimetry and to improve the count statistics of the data. Measured attenuation correction should be employed. The image should be reconstructed with the standard clinical reconstruction including all necessary corrections (random, scatter, attenuation). A set of calibration phantoms including at the minimum the Hoffman brain phantom and the uniform cylinder should be run periodically to assess scanner stability (qualitative and quantitative, resp.). 

Subject conditions during the performance of PET scans should be fully characterized and standardized whenever possible. PET studies should be performed during “a resting state” (e.g., eyes open, ears unoccluded in a dark room with minimal ambient noise). Procedures to minimize head movement during scans should be implemented using well-tolerated head immobilization procedures. The use of medications and the behavioral state of patients at the time of the scan also should be carefully considered.

### 6.3. F-18 FDG PET Utility in Epilepsy

**Figure 6:**
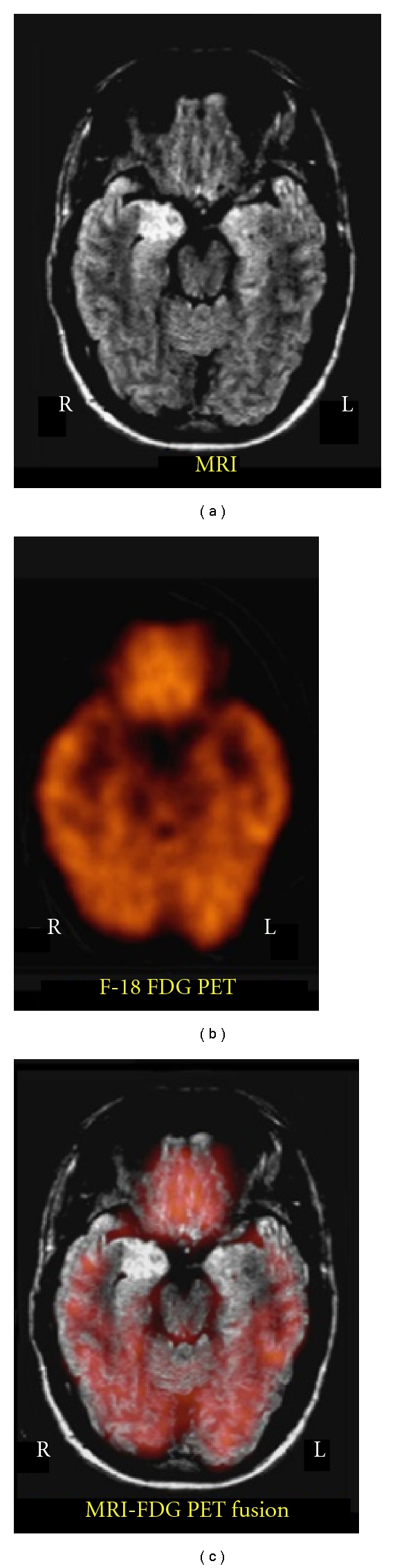
16-year-old boy with temporal lobe epilepsy and hippocampal sclerosis in the right mesial temporal lobe on MRI. (a) MRI shows abnormal high signal intensity in the right mesial temporal lobe (hippocampal region). (b) FDG PET scan shows a focal reduction of FDG uptake in the right mesial temporal lobe (hippocampal region). (c) MRI-PET fusion image illustrating that the reduction in FDG corresponds to the region of MRI increase in signal intensity.

F-18 FDG PET is typically seen in patients with hippocampal sclerosis on MRI. This is illustrated in Figure 6 in a 16-year-old boy with temporal lobe epilepsy and hippocampal sclerosis in the right mesial temporal lobe on MRI. The MRI shows abnormal high signal intensity in the right hippocampal region. The FDG PET shows a corresponding area of focal reduction of FDG uptake in the right hippocampal region. After left temporal lobectomy, the patient was rendered seizure free. 

**Figure 7:**
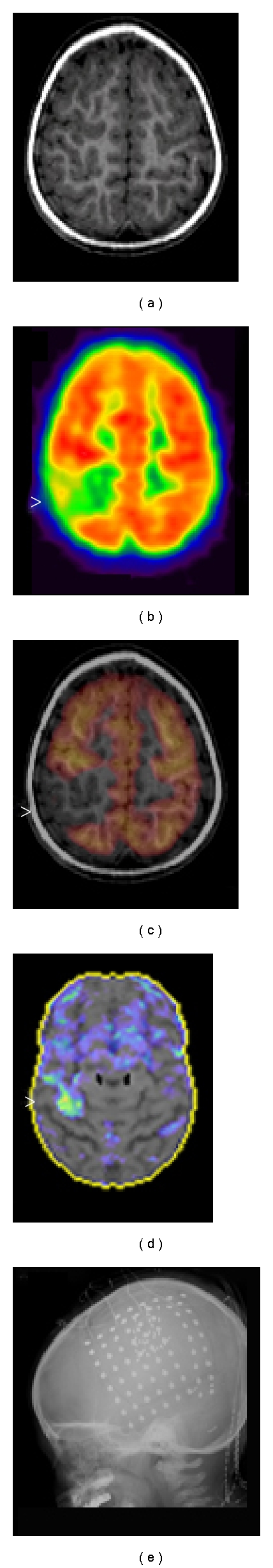
Two-year-old female with intractable partial epilepsy and developmental delay. (a) MRI is normal. (b) Focal F-18 FDG reduction in the right superior parietal lobe representing the epileptogenic focus (arrowhead). (c) MR-PET fusion image. Focal FDG reduction in the right superior parietal lobe representing the epileptogenic focus (arrowhead). (d) 3D-SSP map shows significant reduction of F-18 FDG (arrowhead). (e) Placement of intracranial electrodes guided by F-18 FDG PET is shown on skull radiograph.

Presurgical evaluation and the surgical treatment of nonlesional neocortical epilepsy is one of the most challenging areas in epilepsy surgery. Fluorodeoxyglucose (FDG) PET shows hypometabolism in a majority of patients with nonlesional temporal lobe epilepsy (TLE), even in the absence of hippocampal atrophy. In extratemporal epilepsy where the MRI scan is normal, there is a less likely chance to find the epileptogenic focus by F-18 FDG PET hypometabolism alone.  Figure 7 shows an example where the MRI was normal in a 2-year-old female with intractable partial epilepsy and developmental delay where the F-18 FDG PET unequivocally identified the epileptogenic focus. EEG showed frequent and focal discharges from the right central parietal region. In this case the F-18 FDG localized the epileptogenic region suitable for grid placement and invasive intracranial EEG monitoring. 

**Figure 8:**
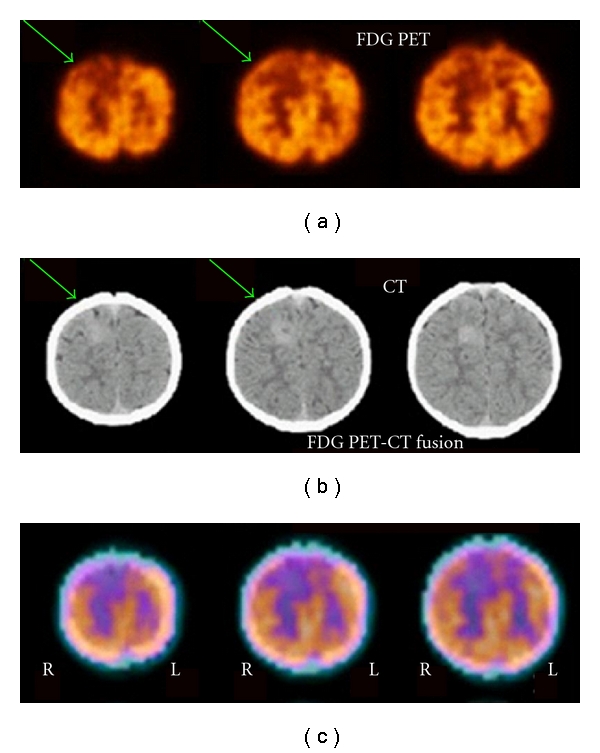
F-18 FDG PET findings in a 1-year-old boy with tuberous sclerosis. (a) FDG PET portion of PET-CT scan showed reduced metabolism in the multiple areas of the tubers, with significant reduction in the focus in the right parasagittal frontal lobe (arrow). (b) CT portion of PET-CT scan showing sclerosis in the region of the right parasagittal frontal lobe tuber (arrow). (c) PET-CT fusion image showing that the areas of decreased metabolism correspond to the prominent right parasagittal frontal lobe tuber.

Interictal FDG PET studies will have limited usefulness in the presence of multiple hypometabolic regions in patients with multifocal brain syndromes, such as in children with tuberous sclerosis. Such children with multifocal lesions represent a special challenge during presurgical evaluation. The goal of functional imaging in these cases is to identify the epileptogenic lesions and differentiate them from nonepileptogenic ones. In this context, ictal rCBF SPECT may have useful clinical applications but may be technically challenging when seizures are short, as is particularly common in frontal lobe epilepsy and in children who have infantile spasms that are associated with multifocal cortical dysplasia [27].  Figure 8 illustrated F-18 FDG PET findings in a 1-year-old boy with tuberous sclerosis who underwent a PET-CT scan. Anatomically there were several lesions that were abnormal even on the CT portion of the PET-CT scan. These areas also showed reduced FDG uptake on PET. Ictal SPECT was able to identify the dominant area of presumed epileptogenesis associated with a large tuber in the right frontal lobe.

### 6.4. Outcome Prediction Using FDG PET

Although FDG PET images can be analyzed visually, additional information can be obtained by semiquantitative analysis, such as left-to-right asymmetry indices. Semiquantitative analysis using the asymmetry index is generally considered significant when a difference of 15% or greater exists between the affected and contralateral sides [28]. Quantitative asymmetry indices should reduce potential error due to the misinterpreting of these normal left-to-right variations [29]. Registration programs can be used to align structural MRI and PET for more precise anatomic localization of the hypometabolic area. 

Although regional hypometabolism is typically present in the temporal lobe ipsilateral to EEG seizure onset, other brain regions may also show patterns of glucose hypometabolism [30]. For example, a FDG PET study of patients with temporal lobe epilepsy demonstrated hypometabolic regions ipsilateral to seizure onset that included lateral temporal (in 78% of patients), mesial temporal (70%), thalamic (63%), basal ganglia (41%), frontal (30%), parietal (26%), and occipital (4%) regions [30]. In pure TLE, however, the extratemporal hypometabolic regions rarely show epileptiform activity on EEG but may be affected by rapid seizure propagation [30]. Unilateral temporal hypometabolism predicts good surgical outcome from temporal lobectomy, and the greater the metabolic asymmetry, the greater the chance of becoming seizure-free [31].

## 7. Multimodal Imaging in Epilepsy

### 7.1. Interictal SPECT versus Interictal PET

FDG PET reflects glucose metabolism of the brain as an indirect measure of neuronal function while SPECT represents cerebral blood flow changes. Although PET has higher spatial resolution and lower background activity, one of the limitations of FDG-PET for the evaluation of epilepsy during the ictal phase is its low temporal resolution. This is due to a tracer uptake period (30–45 minutes) that is significantly longer than the average seizure duration (1-2 minutes), which leads to a mixture of interictal-, ictal-, and postictal-phase images, and this makes only interictal PET feasible. FDG is glucose analogue which is actively transported into metabolically active cells and then temporarily held in the intracellular space due to phosphorylation by hexokinase. However, this is a reversible event, and FDG uptake is both slower and shorter lasting than incorporation of SPECT tracers. Therefore, PET is used to obtain clinical information during the interictal phase. The sensitivity of interictal PET is higher than that of interictal SPECT, as described in a previous meta-analysis by Spencer [32]. One potential explanation for this is the uncoupling of blood perfusion and metabolism, in which there is more reduction in regional cerebral glucose metabolic rates than in regional cerebral perfusion. Evidence for this uncoupling was demonstrated in prior studies using O-15 H2O and F-18 FDG PET [33, 34] and using ratio imaging of interictal Tc-99m HMPAO SPECT divided by F-18 FDG-PET [35, 36].

### 7.2. Ictal SPECT versus Interictal PET

**Figure 9:**
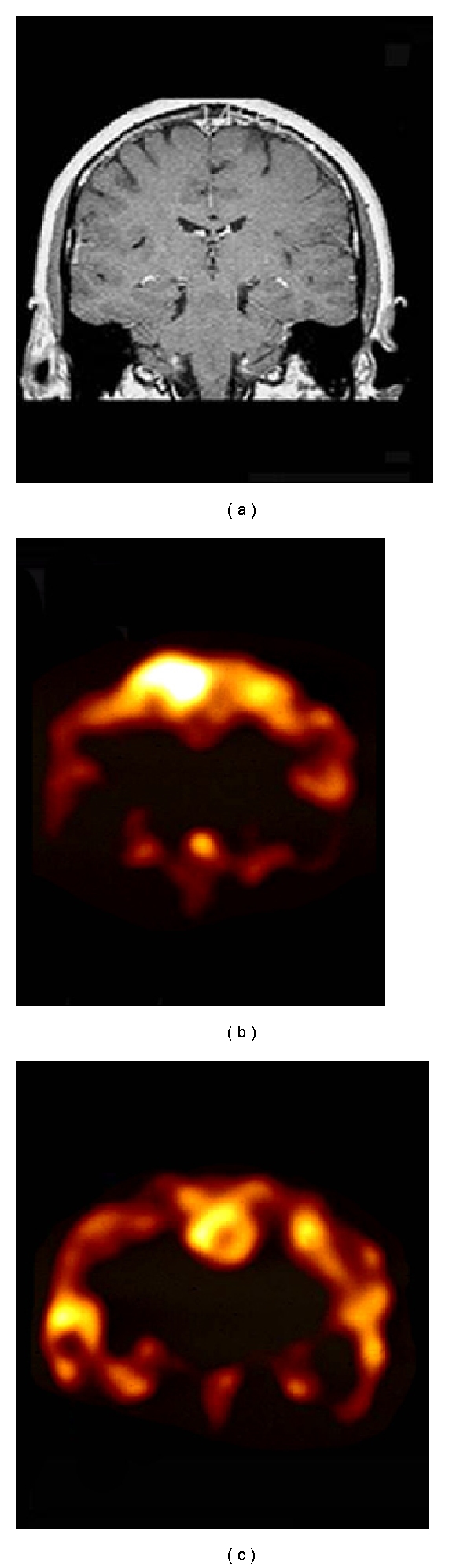
42-year-old female with intractable seizures with seizure activity frequency of 1-2 brief seizures per day, felt to arise from the frontal lobe regions, but were nonlocalizing by video-EEG monitoring. (a) The MRI scan is normal. (b) The ictal Tc-99m HMPAO brain SPECT scan showed a focal area of significant hyperemia in the right mesial frontal lobe. (c) There was minimal reduction of F-18 FDG uptake in this location, but this was not specific for the identification of the epileptogenic focus.

Ictal SPECT and interictal FDG-PET were found to have similar sensitivity [32, 37–39]. Several articles published over the past decades have compared interictal PET and ictal/interictal SPECT. The limitation on the localization capability of interictal FDG PET in extratemporal lobe epilepsy is illustrated by Figure 9 in a case of a 42-year-old female with intractable seizures with seizure activity frequency of 1-2 brief seizures per day, felt to arise from the frontal lobe regions, but were nonlocalizing by video-EEG monitoring. The patient underwent an F-18 FDG brain PET scan which was nonlocalizing. An interictal Tc-99m-HMPAO brain SPECT scan also showed minimal reduction in the frontal lobe, as well as other areas of the brain. An ictal brain SPECT scan showed significant hyperemia in the right frontal lobe. The figure shows a correlative image of the significant hyperemia on ictal SPECT as compared with nonspecific reduction on F-18 FDG PET which is confirmatory but by itself was not localizing. This scan illustrates the relative nonspecificity of mild areas of reduction on F-18 FDG brain PET scan in cases of frontal lobe epilepsy, and in these situations an ictal Tc-99m HMPAO brain SPECT scan can provide greater accuracy in diagnosis. 

**Figure 10:**
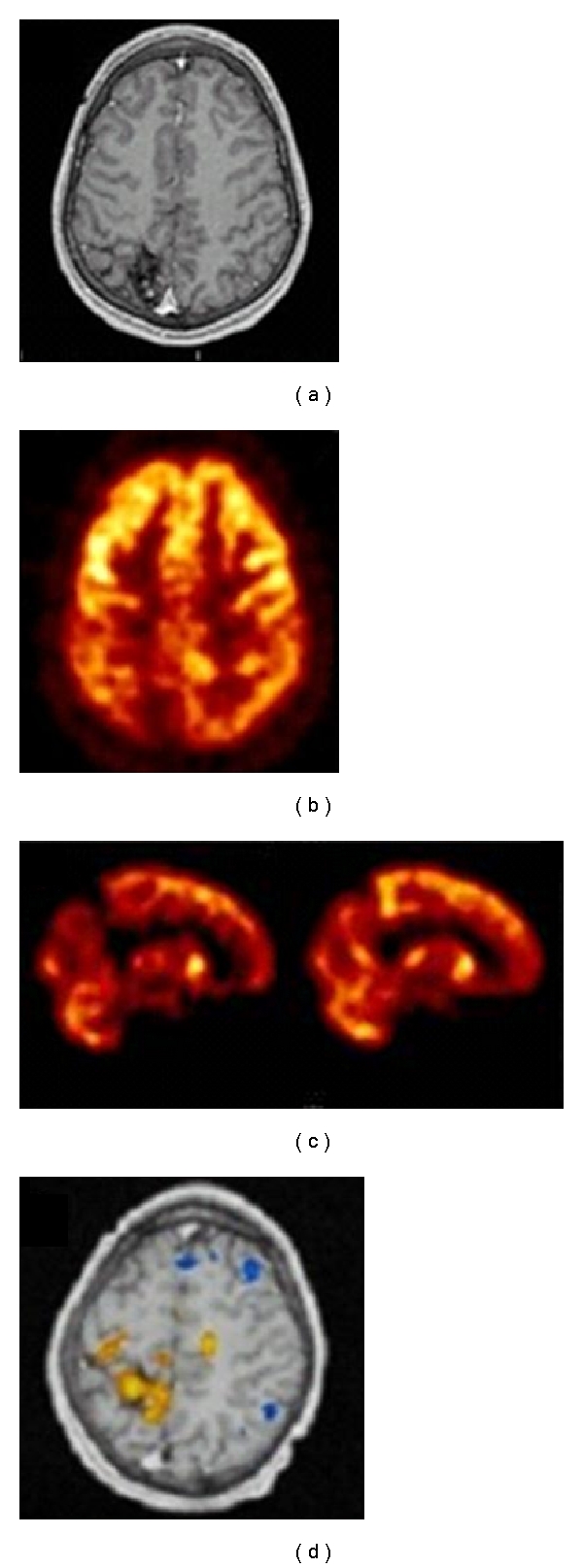
Eleven-year-old male suffering from generalized tonic-clonic seizure disorder. (a) MRI scan of an-11-year old boy suffering from seizure disorder since the age of 7. The patient has tonic-clonic seizures. The MRI scan shows an arterial venous malformation in the right parietal lobe. (b) The interictal brain F-18 FDG PET scan shows an area of reduced metabolic activity in the right parietal lobe consistent with the location of the arterial venous malformation. (c) Images from an interictal Tc-99m ECD brain SPECT (top) as compared to an ictal technetium-99m ECD brain SPECT scan (bottom). One can see significant hyperemia anterior and inferior to the region of the arterial venous malformation on the ictal Tc-99m ECD brain SPECT scan as compared to the interictal brain SPECT scan. (d) SISCOM analysis where the ictal and interictal SPECT are compared and statistically significant differences between the two are mapped onto the patient's MRI scan. One can see significantly increased differences in the ictal study as compared to the interictal study in the anterior region and in the location of the arterial venous malformation (highlighted yellow areas). The highlighted blue area shows areas of decreased uptake on the ictal scan as compared to the interictal scan, which can generally be seen positioned randomly throughout the cortex and do not have clinical or localizing significance.

The importance of precise localization using a statistical analysis method (such as SPM) and an automated registration mapping method (such as AIR) is illustrated in Figure 10 of a in 11-year-old male suffering from generalized tonic-clonic seizure disorder since the age of 7. EEG showed right parietooccipital focal slow and sharp waves. MRI showed right parietooccipital cavernous venous angioma with evidence of bleeding. An interictal SPECT scan shows reduced perfusion in the region of the arterial venous malformation, in addition to a large area around the lesion. An F-18 FDG PET scan also shows reduction around the large AVM area. Ictal SPECT showed hyperperfusion in a region anterior and inferior to the AVM. This is shown on a SPM analysis infusion scan correlating regional cerebral perfusion with a T1-weighted MRI scan. The patient underwent electrocorticography and mapping of the lesion for subsequent resection of the lesion and surrounding epileptogenic area. On electrocorticography and mapping, the area of increased epileptogenesis was found to correlate with the findings of ictal SPECT.

## 8. Summary

The introduction of noninvasive neuroimaging methods, such as single-photon emission computed tomography (SPECT), positron emission tomography (PET), and magnetic resonance imaging (MRI), has dramatically changed presurgical epilepsy evaluation. Previous studies have demonstrated the diagnostic performance of each imaging modality and the value of quantitative analysis of ictal and interictal regional cerebral blood flow SPECT and F-18 FDG PET images. Overall, ictal SPECT has the highest diagnostic sensitivity for both temporal and extratemporal lobe epilepsy, and PET is known to have high sensitivity for the evaluation of extratemporal lobe epilepsy. PET can be employed as a complimentary imaging modality to SPECT in neocortical epilepsy. Quantitative image analysis can further improve diagnostic accuracy of ictal and interictal SPECT and F-18 FDG PET.
